# Genetic susceptibility of hypertension‐induced kidney disease

**DOI:** 10.14814/phy2.14688

**Published:** 2020-12-30

**Authors:** Chao Zhang, Xing Fang, Huawei Zhang, Wenjun Gao, Han Jen Hsu, Richard J. Roman, Fan Fan

**Affiliations:** ^1^ Department of Pharmacology and Toxicology University of Mississippi Medical Center Jackson Mississippi USA; ^2^ Department of Urology Zhongshan Hospital Fudan University Shanghai China

**Keywords:** genetic variants, hypertension, immune cell function, podocytes, renal disease, renal fibrosis

## Abstract

Hypertension is the second leading cause of end‐stage renal disease (ESRD) after diabetes mellitus. The significant differences in the incidence of hypertensive ESRD between different patient populations worldwide and patients with and without family history indicate that genetic determinants play an important role in the onset and progression of this disease. Recent studies have identified genetic variants and pathways that may contribute to the alteration of renal function. Mechanisms involved include affecting renal hemodynamics (the myogenic and tubuloglomerular feedback responses); increasing the production of reactive oxygen species in the tubules; altering immune cell function; changing the number, structure, and function of podocytes that directly cause glomerular damage. Studies with hypertensive animal models using substitution mapping and gene knockout strategies have identified multiple candidate genes associated with the development of hypertension and subsequent renal injury. Genome‐wide association studies have implicated genetic variants in *UMOD*, *MYH9*, *APOL‐1*, *SHROOM3*, *RAB38*, and *DAB2* have a higher risk for ESRD in hypertensive patients. These findings provide genetic evidence of potential novel targets for drug development and gene therapy to design individualized treatment of hypertension and related renal injury.

## Graphical abstract

GWAS identified sequence variants in several genes could alter genetic susceptibility of hypertension‐induced kidney disease.

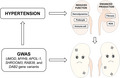

## 1 INTRODUCTION

Chronic kidney disease (CKD) refers to the progressive decline of renal function that results directly from primary renal disorders or is secondary to hypertension, diabetes, and obesity. According to epidemiological reports from the US Centers for Disease Control and Prevention, the prevalence of CKD is 15% in US adults and is expected to continue to rise in the future, which is increasing the burden on the medical care system (CDC, 2019). End‐stage renal disease (ESRD) occurs when the estimated glomerular filtration rate (GFR) falls to less than 10% of normal (Inker et al., 2014). Hypertension is the second leading cause of ESRD after diabetes mellitus. According to the statistical data published by the United States Renal Data System, hypertension accounts for 26% of the patients with ESRD after diabetes, which accounts for 38% (CDC, 2019; Saran et al., 2017). Intriguingly, the incidence of ESRD differs across ethnic backgrounds. Hypertension is listed as the cause of ESRD in 35% of African American patients compared with only 25% of Caucasians in the US (Boone, 2000; Freedman, 2002; Freedman et al., 2005; Martins et al., 2002). More recent studies demonstrated that African Americans are three times more susceptible to renal disease than whites (CDC, 2019), especially associated with hypertension, since they often develop CKD when systemic blood pressure rises to a mild‐to‐moderate range of hypertension at a young age (Hsu et al., 2003; Peralta et al., 2010). Another finding is that hypertensive renal disease occurs at a significantly higher frequency among the first‐ and second‐degree relatives of CKD patients than in individuals without a family history of CKD, suggesting that the susceptibility of renal disease to hypertension segregates in families (Bergman et al., 1996). These findings indicate that the progression to ESRD in hypertension is determined, at least in part, by heritable factors. Renal function often can be altered by changing renal hemodynamics, producing renal reactive oxygen species (ROS), promoting renal fibrosis and inflammation, and diminishing podocyte function in hypertension, diabetes, obesity, and aging (Csipo et al., 2018; Lee et al., 2018; Lv et al., 2018a, 2018b; Mattson, 2014). Regardless of the underlying mechanisms, these studies provide the opportunity to manage the progression of CKD in hypertensive patients by modifying the expression of the causal genes that determine heritable susceptibility. This review summarizes recent findings that highlight the identification of some of the genetic factors underlying the susceptibility of hypertension‐induced renal disease in animal and human studies.

## 2 GENES INVOLVED IN THE REGULATION OF RENAL HEMODYNAMICS

The common outcomes of hypertensive renal disease are albuminuria, glomerulosclerosis, and interstitial fibrosis. Currently, there are several theories concerning how systemic hypertension causes renal injury (Bidani & Griffin, 2004; Hadtstein & Schaefer, 2008; Vaidya & Williams, 2012; Vasavada & Agarwal, 2003). One of the mechanisms involves an impairment of renal autoregulation (Bidani & Griffin, 2004; Carlstrom et al., 2015). Renal autoregulation refers to the intrinsic capacity of the kidney to maintain constant renal blood flow (RBF) and GFR despite fluctuations of systemic blood pressure over a certain range (Navar, 1978). RBF autoregulation is largely mediated by the myogenic response of preglomerular vasculature (Cupples & Loutzenhiser, 1998; Hayashi et al., 1989; Loutzenhiser et al., 2002) and the macula densa (MD)‐related tubuloglomerular feedback (TGF) (Braam et al., 1993; Vallon, 2003), both of which regulate preglomerular vascular tone in response to increased renal perfusion pressure (RPP) (Cupples & Braam, 2007).

Under normal conditions, elevations in systemic pressure are restricted from being transmitted to the glomerular capillaries. Indeed, the high prevalence of hypertension is the major cause of CKD. However, the incidence of CKD is relatively low in the population of hypertensive patients, except in genetic and experimental animal models of hypertension with impaired autoregulation of RBF, as well as in certain racial groups and individuals with a genetic predisposition to renal disease (Hsu et al., 2005; Kopp, 2013). Same as spontaneously hypertensive rat (SHR) and angiotensin II (Ang II)‐dependent models of hypertension, most hypertensive patients exhibit intact RBF autoregulation; they develop benign nephrosclerosis characterized by narrowing of preglomerular arterioles, which limits glomerular injury (Griffin, 2017; Hayashi et al., 1992a; Ren et al., 2010). On the other hand, RBF autoregulation is impaired and elevations in glomerular capillary pressure (Pgc) contribute to the development of proteinuria and glomerulosclerosis in Dahl Salt‐sensitive (Dahl S) (Ge et al., 2014; Ren et al., 2014; Williams, Fan, et al., 2012), Fawn‐Hooded hypertensive (FHH) (Fan, Geurts, et al., 2020; Fan, Gao, et al., 2020), Milan Normotensive (Ge et al., 2017), DOCA‐salt and reduced renal mass hypertensive rats (Griffin, 2017), as well as in some diabetic models (Hayashi et al., 1992b; Kojima et al., 2013). Moreover, servo‐control of RPP has been reported to prevent the infiltration of immune cells and the development of proteinuria and renal injury in Dahl S rats (Evans et al., 2017). Several mechanisms have been identified to explain the link between changes in RBF autoregulation and the development of proteinuria and glomerulosclerosis. Elevations in systemic pressure that increase Pgc and cause capillary distention increase GFR and the filtered load of protein by increasing both the net filtration pressure and filtration area. Abnormal renal autoregulation also fails to buffer elevations in systemic pressure from increasing Pgc. These models also exhibit glomerular hypertrophy and capillary ballooning, capillary unfolding, and microaneurysm formation secondary to mechanical distention, especially at the vascular pole (Kriz et al., 1996; Pavenstadt et al., 2003). Podocytes that support those deranged capillaries are exposed to increased mechanical stress, leading to podocyte injury and detachments from the glomerular basement membrane (GBM). As podocytes are terminally differentiated and cannot proliferate, they are no longer able to cover the increased surface area. This results in further capillary distension until the exposed GBM attaches to the glomerular parietal cells and form a tuft adhesion to Bowman's capsule. These cells proliferate along the exposed GBM, resulting in the collapse of the capillary loop and the initiation of focal glomerular sclerotic lesions (Kriz & LeHir, 2005; Pavenstadt et al., 2003).

### 2.1 Genes involved in the regulation of the myogenic response

The FHH rat is a model of hypertensive nephropathy (van Dokkum et al., 1999; Kuijpers et al., 1986; Simons et al., 1993; Verseput et al., 1998). Roman and his colleagues identified that chromosome 1 is responsible for altered renal autoregulation and proteinuria in FHH rats (Lopez et al., 2006). They then narrowed the region by mapping in overlapping congenic strains. They identified a 2.4‐Mb region of chromosome 1 from the normal Brown Norway (BN) rat onto the homologous region on FHH genetic background. They found improved autoregulation of RBF and attenuated proteinuria in FHH.1^BN^ congenic rats (Burke et al., 2013). Sequence analysis of genes in the 2.4‐Mbp region of chromosome 1 revealed 15 known and predicted genes in the region, of which three genes (γ‐adducin (*Add3*), dual‐specificity phosphatase 5 (*Dusp5*), and X‐prolyl aminopeptidase 1 (*Xpnpep1*)) exhibit sequence variants in the coding regions (Burke et al., 2013). DUSP5 is a member of the threonine/tyrosine phosphatase family with the ability to specifically dephosphorylate the key molecules that regulate intracellular signal transduction, including extracellular signal‐related kinase and protein kinase C (PKC) (Kucharska et al., 2009; Owens & Keyse, 2007; Zhang et al., 2020). A study revealed that knockout (KO) of *Dusp5* enhanced the myogenic response of the middle cerebral artery and autoregulation of cerebral blood flow in FHH rats (Fan et al., 2014). Downregulation of DUSP5 enhanced myogenic reactivity and autoregulation of RBF (Zhang et al., 2019). Proteinuria, glomerular injury, macrophage infiltration, and interstitial fibrosis were reduced after induction of hypertension in this model (Zhang et al., 2019). ADD3 is a membrane cytoskeletal protein that binds with calmodulin and is involved in the spectrin/actin network assembly (Matsuoka et al., 1996, 2000). It serves as a substrate for PKC (Matsuoka et al., 1998, 2000), tyrosine kinase (Matsuoka et al., 2000), and Rho kinase (Kimura et al., 1998). Adducin proteins form heterodimers or tetramers to function, and ADD3 dimerizes with ADD1 in the kidney (Matsuoka et al., 2000). Knockdown of *Add3* by dicer‐substrate short interfering RNA impaired the myogenic response of the renal afferent arteriole (Af‐art) via increased potassium channel function (Fan et al., 2017). Subsequently, Fan et al. rigorously addressed the role of *Add3* in hypertensive renal disease. They found that knock‐in of wild‐type *Add3* in the FHH genetic background reduced hypertensive renal disease by rescuing abnormal renal hemodynamics and lowering Pgc (Fan, Geurts, et al., 2020; Fan, Gao, et al., 2020).

The Dahl S rat is the animal model that mimics salt‐induced hypertension and renal disease in humans. Dahl S rats rapidly develop progressive proteinuria and renal injury after the development of salt‐sensitive hypertension (Fan & Roman, 2017; Hua et al., 1990; Rapp & Dene, 1985; Sterzel et al., 1988). Previous studies on the genetic determinants of the susceptibility to CKD in Dahl S rats have demonstrated that some genes are responsible for the structural and functional changes in preglomerular vasculature and impaired vascular tone (Fan & Roman, 2017; Zhang et al., 2018). 20‐HETE is a metabolite of arachidonic acid formed by cytochrome P‐450 *(Cyp) 4a* genes, all of which are located in a cluster on chromosome 5 in rats (Fan et al., 2015, 2016; Roman, 2002; Zhang et al., 2018). A genetic marker in *Cyp4a2* cosegregated with hypertension in an F2 cross of Dahl S and Lewis rats (Stec et al., 1996). Roman et al. later found that transfer of the *Cyp4a* region of chromosome 5 from Lewis rats increased the renal level of 20‐HETE, improved renal autoregulation and attenuated renal injury in Dahl S rats (Fan & Roman, 2017; Fan et al., 2013; Roman et al., 2006; Williams et al., 2008). Elevations in pressure increase 20‐HETE production in renal and cerebral arteries (Fan et al., 2013; Gebremedhin et al., 2000; Imig et al., 1996; Zou et al., 1996). 20‐HETE enhances the myogenic response by inhibiting the calcium‐sensitive potassium channel activity in renal vascular smooth muscle cells (VSMCs) (Zou et al., 1996), leading to membrane depolarization and enhanced calcium entry via the voltage‐sensitive calcium channels (Gebremedhin et al., 1998). Inhibitors of the synthesis of 20‐HETE block the myogenic response of Af‐art (Ge et al., 2013) and autoregulation of RBF (Zou, Imig, Kaldunski, et al., 1994) both *in vivo* and *in vitro*. These studies revealed that a genetic deficiency in the expression of CYP4A and the production of 20‐HETE is associated with the pathogenesis of hypertensive nephropathy in Dahl S rats.

More recently, the SH2 adaptor protein P66SHC (encoded by *Shc1*) was found to regulate renal vascular tone in hypertensive nephropathy. In Dahl S rats, transgenic upregulation of P66SHC was associated with increased renal injury, whereas the deletion of *P66shc* restored the myogenic response of renal preglomerular arterioles *ex vivo* and promoted contraction of primary VSMCs (Miller et al., 2016). In primary VSMCs, P66SHC restricted agonist‐induced activation of transient receptor potential cation channels to attenuate calcium influx, indicating the mechanism involved in the impairment of renal vascular reactivity (Miller et al., 2016). Although a sequence variant in *Shc1* has yet to be identified in Dahl S rats relative to other strains, these results suggest a role for P66SHC as a potential new candidate pathway that impairs renal vascular tone in Dahl S rats and contributes to hypertensive nephropathy (Miller et al., 2016).

### 2.2 Genes involved in the regulation of the TGF response

TGF response is another indispensable component of renal autoregulation that contributes to the slower restoration of vascular resistance to the increased RPP. Abolishing the MD‐TGF response attenuated the autoregulation of single nephron GFR and Pgc during changes in RPP (Carlstrom et al., 2015). MD cells, as a sodium sensor, are the major contributor regulating the TGF response and release of renin from the juxtaglomerular cells. Stimulation of the MD cells by an increased sodium concentration enhances the production of ATP/adenosine and intracellular calcium, resulting in the constriction of the Af‐art. In this regard, RBF was poorly autoregulated in adenosine A1 receptor (*Adora1*)‐deficient mice (Hashimoto et al., 2006; Just & Arendshorst, 2007). Roman and his colleagues reported that administration of ADORA1 agonist reduced the inner diameter of the Af‐art in rats (Ge et al., 2014). The ATP‐gated P2X receptor cation channel family also plays a role in regulating the TGF response, evidenced by studies in P2X1 receptor KO mice (Inscho et al., 2003). Extraglomerular mesangial cells that are localized between the MD and Af‐art also play a vital role in regulating the TGF response, possibly involving communication between VSMCs and gap junctions (Carlstrom et al., 2015; Ren et al., 2002). Gap junction proteins, such as connexin 40, have been reported to play a permissive role in the TGF response (Just et al., 2009). Additionally, a further study by the Roman group showed that the myogenic response and TGF of Af‐art were impaired in Dahl S rats, suggesting that 20‐HETE may contribute to the TGF response (Ge et al., 2014). 20‐HETE inhibitors and antagonists also block the TGF response of both *in vivo* and *in vitro* by attenuating the vasoconstrictor response of the Af‐art to adenosine released by the MD (Ge et al., 2013; Zou, Imig, Ortiz de Montellano, et al., 1994). More recently, evidence in mice demonstrated that the expression of MD nitric oxide synthase‐1β (NOS‐1β) was enhanced on a high salt diet, suggesting NOS‐1β may contribute to the downregulation of the TGF response and the development of salt‐sensitive hypertension (Lu et al., 2016).

## 3 GENES INVOLVED IN THE REGULATION OF RENAL ROS PRODUCTION

Increased ROS production in the renal outer medulla is another important mechanism that contributes to hypertension and renal injury in Dahl S rats (Cowley et al., 2015; Mori et al., 2007). ROS are a series of oxygen‐derived reactive molecules, including superoxide, hydrogen peroxide (H_2_O_2_), and nitric oxide, typically produced by NAD(P)H oxidases (NOX) in mitochondria (Boveris & B. Chance, 1973). In the renal medulla, ROS is mainly produced by medullary thick ascending limb (mTAL) of Henle's loop (Gill & Wilcox, 2006; Taylor et al., 2006; Zou et al., 2001). In Dahl S rats, elevated salt intake increases the production of ROS by enhancing the delivery of sodium and water flow to the mTAL, which increases sodium transport and causes a higher energy utilization and mitochondrial oxygen consumption (Cowley et al., 2015). Under normal conditions, the excess production of superoxide and H_2_O_2_ in the mTAL does not diffuse to the surrounding vasa recta. However, an increase in oxygen‐free radicals diffuses to the surrounding vasa recta in Dahl S rats, in which redox balance is shifted toward greater production of ROS, results in a reduction in medullary blood flow (MBF) via the mechanism known as tubular‐vascular cross‐talk (Mori et al., 2007). The fall in MBF and an increase in sodium transport in the mTAL promotes tissue hypoxia and ultimately causes renal interstitial fibrosis and salt‐sensitive hypertension (Mori et al., 2007). For many years, Dr. Cowley and his colleagues have been working on identifying candidate genes that account for the increase in ROS production in the mTAL of Dahl S rats fed a high‐salt diet. They studied the genetic basis of salt‐sensitive hypertension and renal injury by the substitution of BN chromosome 13 into the Dahl S genome and found that hypertension and albuminuria were attenuated in SS.13^BN^ congenic strains (Cowley et al., 2001; Lu et al., 2010; Moreno et al., 2007). The results of cDNA microarrays indicated that genes located on chromosome 13, F5, *Serpinc1*, *Slc19a2*, were differentially expressed in SS.13^BN^ and Dahl S rats when fed a 4% salt diet (Liang et al., 2002, 2008). Genetic substitution mapping using a series of SS.13^BN^ consomic strains identified a region in which ROS production in the mTAL was normalized as compared with Dahl S rats, indicating that the genes responsible for the increased medullary oxidative stress were also located on chromosome 13 (Taylor & Cowley, 2005).

NOX2 is one of the most abundant NOX in the kidney that contributes to hypertension, renal oxidative stress, and injury in Dahl S rats. The *P67phox* subunit of *Nox2* is one of the genes on chromosome 13 that is essential for the activation of NOX2. The congenic SS.13^BN^ rats exhibited significantly lower levels of *P67phox* mRNA, protein, and NOX enzyme activity in the outer medulla compared with Dahl S rats (Zheleznova et al., 2016). The expression of P67PHOX was increased in Dahl S rats, but not in SS.13^BN^ rats, following a high‐salt diet associated with increased mTAL luminal flow (Feng et al., 2012). Introgression of the salt‐resistant *P67phox* allele from the BN rat into the Dahl S genetic background reduced salt‐induced hypertension and renal injury (Zheleznova et al., 2016). These studies have established a role for changes in P67PHOX in altering renal function in Dahl S versus chromosome 13 congenic rats; however, sequencing studies to date have failed to identify any causal variants that affect the expression of P67PHOX and/or the production of basal or stimulated ROS in these strains.

While the previous studies focused on the role of *P67phox* or neutrophil cytosolic factor 2 (*Ncf2*) in the development of hypertension because it is located on chromosome 13, it is important to recognize that both *P67phox* and *P47phox* (*Ncf1*) play a critical role in the activation of ROS in macrophages (Zhong et al., 2018). Indeed, Holmdahl et al. have identified polymorphisms in *Ncf1* and *Ncf2* in rats and mice that lower the formation of ROS and reduce immune cell function (Holmdahl et al., 2016). Deficiency of *Ncf1* has a major effect on chronic inflammatory autoimmune diseases with the surprising observation that a lower reactive oxygen production led to more severe diseases. These findings suggest that the link between *P67phox* and the development of hypertension and renal disease in Dahl S rats might be related to changes in immune cell infiltration.

Other studies have focused on the role of *Nox4* in promoting medullary oxidative stress in Dahl S rats. These studies indicated that elevated expression of NOX4 largely accounts for the excess production of H_2_O_2_ in the mTAL of Dahl S rats (Cowley, Yang, Zheleznova, et al., 2016; Saez et al., 2018; Zheleznova et al., 2016). NOX5 plays an important role in the development of diabetic nephropathy. Since the *NOX5* gene is absent in rodents, the studies regarding NOX5 were conducted in patients and genetically humanized mouse models. The podocyte‐specific human *NOX5*‐transgenic mice exhibited early‐onset albuminuria, podocyte structure changes, and increased systolic blood pressure, associated with the elevated production of ROS (Holterman et al., 2014). In human kidney biopsies, NOX5 was found to be expressed in glomeruli, especially in podocytes and mesangial cells, which appeared to be increased in diabetes (Jha et al., 2017). Overall, these findings have revealed that the genes encoding P67PHOX/P47PHOX/NOX2, NOX4, and NOX5 contribute to hypertension‐ and diabetes‐induced renal injury by altering the renal production of ROS (Table 1).

**Table 1:** Genes recently linked to hypertension‐induced renal disease in human and rodent studies.

Gene	Location	Species	Mechanisms	Reference
*Add3*	Chr.1 (1q55)	Rats (FHH)	Hemodynamics	Fan, Geurts, et al., 2020; Fan et al., 2017; Fan, Gao, et al., 2020
*APOL1*	Chr.22 (22q12)	Human (GWAS)	Podocyte and glomerular function	Friedman & Pollak, 2020; Foster et al., 2013; Genovese, Friedman, et al., 2010; Hayek et al., 2017; Parsa et al., 2013
		*APOL1* transgenic mice	Podocyte and glomerular function	Beckerman et al., 2017; Kopp et al., 2011; Madhavan et al., 2011
*Cyp4A*	Chr.5 (5q35)	Rats (Dahl S, Lewis)	Hemodynamics and sodium transport	Zhang et al., 2019
*DAB2*	Chr.5 (5p13)	Human (GWAS)	?	Qiu et al., 2018; Nair & Kretzler, 2019
*Dab2*	Chr.15 (A1)	Mice	Renal fibrosis	Qiu et al., 2018
*Dusp5*	Chr.1 (1q55)	Rats (FHH)	Hemodynamics	Fan et al., 2014; Zhang et al., 2019
*MYH9*	Chr.22 (22q12)	Human (GWAS)		Bostrom et al., 2010; Freedman et al., 2009; Kao et al., 2008; Liu et al., 2016
*Ncf1 (P47phox)*	Chr.12(12q12)	Rats (Dahl S)	ROS production and immune cell infiltration	Holmdahl et al., 2016; Zhong et al., 2018
*Ncf2 (P67phox)*	Chr.13(13q21)	Rats (Dahl S)	ROS production	Feng et al., 2012; Zheleznova et al., 2016; Zhong et al., 2018
*Nox4*	Chr.1 (1q32)	Rats (Dahl S)	ROS production	Cowley, Yang, Zheleznova, et al., 2016; Saez et al., 2018; Zheleznova et al., 2016
*NOX5*	Chr.15 (15q23)	Mice (humanized)	ROS production	Holterman et al., 2014; Jha et al., 2017
*Pappa2*	Chr.13 (13q22)	Rats (Dahl S)		Cowley, Yang, Kumar, et al., 2016
*RAB38*	Chr.11 (11q14)	Human(GWAS)	Within loci associated with albuminuria	Teumer et al., 2016
*Rab38*	Chr.1 (1q32)	Rats (FHH)	Re‐uptake tubular protein	Rangel‐Filho et al., 2013; Rangel‐Filho et al., 2005
*Shc1* (p66*Shc*)	Chr.2 (2q34)	Rats (Dahl S)	Hemodynamics	Miller et al., 2016
*SHROOM3*	Chr.4 (4q21)	Human (GWAS)	Within eGFR‐related loci	Boger et al., 2011; Kottgen et al., 2009; Prokop et al., 2018; Yeo et al., 2015
*Shroom3*		*Shroom ^null^* mice	Podocyte function	Khalili et al., 2016; Yeo et al., 2015
	Chr.14 (14q22)	Rats (FHH)	Podocyte and glomerular function	
*Tgf‐β1*	Chr.1 (1q21)	Rats (Dahl S)	Renal fibrosis	Chen et al., 2013; Dahly et al., 2002
*Trpc6*	Chr.8 (8q11)	Rats (Dahl S) and mice	Podocyte function and ROS production	Ilatovskaya et al., 2018; Ma et al., 2019; Spires et al., 2018; Staruschenko et al., 2019;Wang et al., 2020
*UMOD*	Chr.16(16p12)	Human(GWAS)		Gudbjartsson et al., 2010; Kottgen et al., 2010; Liu et al., 2011; Rampoldi et al., 2011; Steubl et al., 2019
*Umod*		*Umod* transgenic mice	Sodium transport, ER stress, and apoptosis	Johnson et al., 2017; Trudu et al., 2013

Abbreviations: *Add3*, γ‐adducin; *APOL1*, apolipoprotein L1; Chr., chromosome; *Cyp4A*, cytochrome P‐450 4A; *DAB2*, Disabled homolog 2; Dahl S, Dahl salt‐sensitive; *Dusp5*, dual‐specificity phosphatase 5; eGFR: estimated glomerular filtration rate; ER, endoplasmic reticulum; FHH, Fawn‐hooded hypertensive; GWAS, genome‐wide association study; *MYH9*, myosin heavy chain 9; *Ncf*, neutrophil cytosolic factor; *Nox*, NADPH oxidases; *Rab38*: ras‐related protein 38; ROS, reactive oxygen species; *SHROOM3*: shroom family member 3; *Tgf*‐*β1*, transforming growth factor‐β1; *Trpc*, transient receptor potential canonical.

*Pappa2* is another gene located on chromosome 13 that can protect against salt‐induced hypertension in Dahl S rats. PAPPA2, localized in the cytosol of the epithelial cells of the cortical thick ascending limbs, was differentially expressed between Dahl S rats and SS.13^BN^ when challenged with a high salt diet (Cowley, Yang, Kumar, et al., 2016). However, further study on the knockout or transgenic rats is needed to examine the role of *Pappa2* in affecting sodium homeostasis and salt‐sensitive hypertension.

## 4 GENES INVOLVED IN THE ALTERATION OF IMMUNE CELL FUNCTION

A large body of evidence from humans and animals indicates that immune mechanisms contribute to the development of hypertension and kidney damage. However, the cause‐and‐effect relationship between immunity, hypertension, and renal disease is only beginning to be understood (Mattson, 2019; Rudemiller & Mattson, 2015). Previous studies have documented that hypertensive patients, particularly those with salt‐sensitive hypertension, exhibit an increased infiltration of macrophages and T lymphocytes into the kidney. This infiltration occurs adjacent to damaged glomeruli and tubules, and the degree of infiltration correlates with the severity of tissue damage (Johnson et al., 2005; Mattson, 2014; Olsen, 1972; Sommers et al., 1958). Similarly, a large number of pharmacologic and genetic manipulations have clearly demonstrated the importance of the immune system in the pathogenesis of hypertension and/or renal disease in many different rat models (Mattson, 2019).

Whole genome‐wide association studies (GWAS) have now identified more than 900 genes for hypertension (Evangelou et al., 2018) and a large number of susceptibility loci for renal disease on nearly every chromosome (Gorski et al., 2015; Kottgen et al., 2010). Genetic mapping studies using rat strains that are susceptible (FHH, Dahl S, Buffalo, Munich Wistar Furth, and Stroke‐Prone SHR) and resistant (SHR, BN, Wistar Kyoto, Lewis, August Copenhagen) to renal disease have identified many other genomic regions associated with glomerular injury, proteinuria, and renal interstitial fibrosis (Schulz & Kreutz, 2012). However, the causal variants for most candidate genes in the regions of interest have not been identified or validated, and the mechanisms involved are largely unknown.

Recently, some of the inflammatory genes nominated by human linkage and association studies were evaluated for hypertension and kidney disease using KO studies in Dahl S rats. These genes include *Sh2b3* (also known as *Lnk*), which encodes SH2B adaptor protein 3, involved in cytokine signaling; *Cd14* involved in activation of macrophages and monocytes; and *Cd247*, which encodes for a subunit of CD3 involved in the assembly and expression of the T‐cell receptor complex and signal transduction following binding of antigens (Mattson, 2019). KO of *Cd247* in Dahl S rats reduced infiltration in the number of CD3+ T cells by 90% in the kidney, and the development of hypertension and renal injury (Rudemiller et al., 2014). Similar results were seen in *Sh2b3* KO Dahl S rats (Rudemiller et al., 2015). These studies confirmed that loss of function of these genes could play a role in the development of hypertension in man and renal disease in man and Dahl S rats. However, it is important to recognize that the causal variants in these candidate genes in man remain to be identified and that mutations in these genes have yet to be genetically linked to the development of hypertension or renal disease in Dahl S rats or other models of hypertension.

More recently, the Doris team used another approach and established a genetic link between changes in the immune system and increased susceptibility to renal disease in the Stroke‐Prone SHR (SpSHR) (Dhande et al., 2018). They recognized that the renal disease‐susceptible SpSHR is 87% genetically identical to disease‐resistant SHR and only differ in distinct haplotype blocks in 121 regions of the genome. They identified regions of chromosomes 6 and 17 linked to proteinuria and renal disease in an F2 cross of SpSHR versus SHR (Bell et al., 2011; Braun et al., 2014; Roman & Fan, 2018). The highly polymorphic 9 Mb haplotype region of chromosome 6 harbors 230 IgH genes, of which 49% exhibited nonsynonymous variations in SpSHR and SHR (Braun et al., 2014; Dhande et al., 2018). The increased renal injury in SpSHR was ameliorated by immunosuppression (Braun et al., 2014). The Doris team confirmed that the difference in IgG subtypes that differ in SpSHR versus SHR was normalized using a chromosome 6 congenic strain (Dhande et al., 2018). They concluded that there are one or more genes in the IgH region of chromosome 6 likely influence renal injury by altering B‐ and T‐cell function and the immune response. In subsequent studies, they identified a truncation in another gene (*Stim1*) in the SpSHR rats that contribute to the susceptibility to renal injury (Dhande et al., 2020). STIM1 is an endoplasmic reticulum (ER) calcium sensor that plays a central role in lymphocyte effector and regulatory function. Congenic replacement of truncated *Stim1* with the wild‐type allele restored a major defect in immune function in SHR‐A3, a line of SpSHR, and the ability of hypertension to elicit damage to the kidney was reduced in this model (Dhande et al., 2020). These results suggest that the major immune phenotype produced by the *Stim1* mutation participates in renal injury in SHR‐A3, and this may include injury contributed by autoantibody formation that interferes with T‐cell signaling to program antibodies producing by B cells.

There are several other genes, such as apolipoprotein l1 (*APOL1*) and uromodulin (*UMOD*), also play a role in immune response in hypertensive renal injury, which we will discuss in the latter GWAS section.

## 5 GENES INVOLVED IN THE DEVELOPMENT OF RENAL FIBROSIS

Genes involved in the development of renal fibrosis have been extensively studied in Dahl S rats (Lv et al., 2018a, 2018b). The expression of transforming growth factor‐β1 (TGF‐β1) is a major factor that drives renal fibrosis. Activation of the TGF‐β1 signaling pathway results in the transformation of epithelial cells to myofibroblasts, excess production of extracellular matrix (ECM), and inhibition of ECM degradation (Eddy, 2000; Meng et al., 2015, 2016). The expression of TGF‐β1 is elevated in Dahl S rats fed a high salt diet. Heterozygous knockout of the *Tgf*‐*β1* gene in Dahl S rats reduced proteinuria, glomerulosclerosis, and renal interstitial fibrosis but did not affect blood pressure (Chen et al., 2013). Moreover, chronic administration of an anti‐TGF‐β1 antibody reduced blood pressure and renal injury (Dahly et al., 2002). Tumor necrosis factor‐α (TNF‐α) is a major pro‐inflammatory cytokine that mediates increases in blood pressure (Ramseyer & Garvin, 2013). Liang's group reported that the abundance of TNF‐α and its receptor was found in the kidney of Dahl S rats fed a high‐salt diet, in association with exacerbated glomerulosclerosis and interstitial fibrosis (Huang et al., 2016). Matrix metalloproteinase‐9 and‐2 (MMP‐9, MMP‐2) are enzymes in the gelatinase family that play an important role in the degradation of ECM. The Roman and Williams groups reported that KO of *Mmp2* and *Mmp9*, respectively, on the Dahl S genetic background protected against the development of hypertension and renal interstitial fibrosis when fed a high‐salt diet (Slaughter et al., 2012; Williams, Slaughter, et al., 2012; Zhang et al., 2017). The results suggest that the upregulation of the expression of TGF‐β1, TNF‐α, MMP‐2, and MMP‐9 contributes to the development of hypertension, proteinuria, and glomerular and tubular injury in Dahl S rats. However, the sequence variants of *TGF*‐*β1*, *TNF*‐*α*, *MMP*‐*2*, or *MMP*‐*9* linked to the development of CKD in humans have not been identified.

## 6 GENES INVOLVED IN THE ALTERATION OF PODOCYTE FUNCTION

African Americans have a significantly higher risk of hypertensive and diabetic nephropathy. Numerous studies have sought to identify the genetic variants differentially expressed in African Americans. *APOL1* is one of the most widely examined genes that is associated with hypertension‐induced renal injury. APOL1 is a lytic factor in human serum, which protects against trypanosomes that induce sleeping sickness (Cooper et al., 2017). The two disease‐causing *APOL1* genetic variants that arose in African populations conferred enhanced protection against the virulent subspecies of trypanosomes. In 2010, Giulio Genovese and his colleagues reported that two independent sequence variants G1 and G2 in *APOL1* were associated with a higher risk of developing hypertension‐induced kidney disease in an African‐American population than in the patients of European descent. The latter carries the wild‐type *APOL1* gene G0 (Genovese, Friedman, et al., 2010). They also examined the effects of *APOL1* variants on the progression of hypertension‐induced CKD in large cohorts of black and white patients with CKD attributed to hypertension (Foster et al., 2013; Parsa et al., 2013). They reported the significant association between the risk variants G1 and G2 in *APOL1* and higher rates of ESRD and CKD in black patients (Foster et al., 2013; Parsa et al., 2013). *APOL1* variants‐related hypertensive kidney injury may involve alterations in podocyte function. Although the underlying mechanisms remain to be determined, *APOL1* variants may create pores in cell membranes and injure podocyte function much the same as it lysis trypanosomes (Friedman & Pollak, 2020). Foot process effacement and glomerulosclerosis have been found in transgenic mice that contain podocyte‐specific *Apol* risk alleles, which may be due to alterations in endosomal trafficking and autophagy in podocytes (Beckerman et al., 2017; Kopp et al., 2011; Madhavan et al., 2011). Additionally, *APOL1* increases the vulnerability of podocytes to injury in response to oxidative stress associated with hypertension. *APOL1* risk variants have also been shown to synergistically act with plasma soluble urokinase plasminogen activator receptor (suPAR) levels in the development of podocyte injury and CKD in humans (Hayek et al., 2017).

The recent findings indicate that the genes expressing transient receptor potential canonical (TRPC) channels may also be involved in the podocyte structure and function. The expression and activity of TRPC6 channels were elevated in various *in vivo* and *in vitro* diabetic nephropathy models (Spires et al., 2018; Staruschenko et al., 2019). The elevated TRPC6 channel activity in diabetic kidney disease increases calcium influx into the podocytes, leading to its dysfunction and breakdown of glomerular filtration barrier, and by facilitating the formation of ROS (Staruschenko et al., 2019). These events have been reported involving several mechanisms, including NOX4/TRPC6 (Ilatovskaya et al., 2018), Ang II/TRPC6/NFAT (Ma et al., 2019) pathways, and the rearrangement of the actin cytoskeleton in the podocytes (Wang et al., 2020) in diabetic nephropathy. The podocyte dysfunction in kidney diseases also implicates another TRPC family member, TRPC5. However, results from previous reports have not consistently indicated whether and how TRPC5 plays a role in the development or protection of kidney disease. The inhibition of TRPC5 attenuated progressive kidney injury in AT1R transgenic mice, Dahl S rats (Wang & Reiser, 2018). On the other hand, overexpression or activation of TRPC5 in mice models did not promote renal injury (Wang et al., 2018).

## 7 GENES ASSOCIATED WITH RENAL DISEASE IN GENOME‐WIDE ASSOCIATION STUDIES

GWAS are the human population‐based investigations to search for single nucleotide polymorphisms (SNPs) that occur in a distinct frequency in the patients with a particular disease compared with nonpatient population. During the past decades, the results of a number of important large‐scale GWAS have greatly improved our understanding of the genetic susceptibility to CKD (Wuttke & Kottgen, 2016). More than 50 genetic variants have been identified that are potentially associated with the decline of kidney function and the development of hypertension and renal injury in various studies, among which the most frequently identified gene variants are *UMOD*, *MYH9*, *APOL1*, *SHROOM3*, *RAB38*, and *DAB2*.

Uromodulin encoded by the *UMOD* gene is the most abundant protein excreted in the urine and is exclusively produced by the kidney tubule under normal conditions (Devuyst & Pattaro, 2018). Rare mutations in UMOD can cause autosomal dominant tubulointerstitial kidney diseases (Kemter et al., 2013; Kumar & Muchmore, 1990; Pennica et al., 1987). Recently, GWAS identified *UMOD* variants as a risk factor for CKD and hypertension and suggested that the level of UMOD in the urine could represent a useful biomarker for the progression of CKD to ESRD (Gudbjartsson et al., 2010; Kottgen et al., 2010; Liu et al., 2011; Rampoldi et al., 2011; Steubl et al., 2019). A study demonstrated that the activation of renal sodium cotransporter NKCC2 in transgenic mice potentially links the *UMOD* variants and hypertension (Trudu et al., 2013). Another study revealed that UMOD‐producing tubular cells were associated with early activation of ER stress pathway, innate immune mediators, and apoptotic signaling (e.g., caspase‐3), as well as the deficiency of autophagy (Johnson et al., 2017). Human cells producing mutant UMOD were susceptible to TNF‐α‐ and TRAIL‐mediated apoptosis due to increased expression of the ER stress mediator tribbles‐3 (Johnson et al., 2017).

Nonmuscle myosin IIA (*MYH9*) has been reported to be associated with the increased susceptibility to hypertension and related CKD progression to ESRD in African Americans and a Chinese population (Freedman et al., 2009; Kao et al., 2008; Liu et al., 2016). A GWAS in 1,000 diabetic African Americans with ESRD in comparison with 500 nondiabetic cases and 500 non‐nephropathy controls demonstrated that 16 SNPs in pooled DNA were associated with nondiabetic ESRD and 12 of these SNPs are in or near the *MYH9* gene (Bostrom et al., 2010). In addition, *MYH9* polymorphisms also have been associated with the increased risk of HIV‐associated nephropathy and focal segmental glomerulosclerosis in African‐Americans (Genovese, Tonna, et al., 2010; Kopp et al., 2008; Winkler et al., 2010; Zhou et al., 2011).

*MYH9* was initially identified as a candidate gene for CKD in GWAS studies on chromosome 22 (22q12). However, despite intensive efforts, no suggested functional mutations have been identified in this gene. Subsequent studies identified closely linked mutations in *APOL1* as the likely causal variants linking the same locus on chromosome 22 with CKD in African descent populations (Hawkins et al., 2015; Tzur et al., 2010). The strongest associations have been observed with focal segmental glomerulosclerosis (FSGS), human immunodeficiency virus‐associated nephropathy, and hypertension‐induced FSGS. The odds ratios for the APOL1 G1 (non‐synonymous coding variant 342G∶384 M) and G2 (6 basepair deletion) range from 10.5 for FSGS to 7.3 in nondiabetic ESRD (Freedman et al., 2011). As discussed earlier, the APOL1 gene is the most widely studied gene linked to ESRD, and its mechanism involves alterations in podocyte function.

Shroom family member 3 (*SHROOM3*) gene has been associated with CKD in multiple GWASs (Boger et al., 2011; Kottgen et al., 2009; Prokop et al., 2018; Yeo et al., 2015), but the overall contribution of these noncoding and rare variants to CKD risk is very modest (Boger & Heid, 2011). SHROOM3 is an actin‐binding protein, and its role in kidney development and function has been investigated in animal models. Transferring a 6.1‐Mb region of chromosome 14 containing the wild‐type *Shroom3* gene from BN to FHH rat improved glomerular function in the FHH rat (Yeo et al., 2015). The wild‐type *Shroom3* allele, but not the FHH *Shroom3* allele, rescued the glomerular defects, suggesting that the FHH *Shroom3* allele was defective and likely contributed to renal injury in this model of CKD (Yeo et al., 2015). Thirteen amino acid variants were identified in the FHH SHROOM3 protein compared with the BN rat. Six of these 13 variants were predicted to damage to SHROOM3 function, and several variants localize to the actin‐binding region that could affect podocyte structure and function. However, the causal variants in the FHH rats have not been identified or validated. *Shroom3* null mice showed marked glomerular abnormalities and the disruption in podocyte arrangement and morphology (Khalili et al., 2016). *Shroom3* heterozygous mice developed podocyte irregularities that manifested as adult‐onset glomerulosclerosis and proteinuria (Khalili et al., 2016). These podocyte‐specific abnormalities were associated with altered Rho‐kinase/myosin II signaling and loss of apically distributed actin (Khalili et al., 2016). Further study on CKD patients was implemented to characterize coding and noncoding variants of human *SHROOM3* risk locus (Prokop et al., 2018). The high‐effect alleles include a coding variant (P1244L), which attenuates the interaction of *SHROOM3* with Protein 14–3–3 (a chaperone‐like serine/threonine‐binding protein that can alter the structure or function of target proteins) (Faul et al., 2008; Prokop et al., 2018).

*RAB38* gene variants influence albuminuria in patients of European ancestry with diabetes (Teumer et al., 2016). This finding is consistent with previous studies in FHH rats, indicating that *Rab38* contributes to hypertension‐associated kidney disease (Rangel‐Filho et al., 2005, 2013). The FHH rat was shown to be a natural *Rab38* knockout animal, and transgenic *Rab38* rats on the FHH background lowered 75% albuminuria (Rangel‐Filho et al., 2013). Knockout of the *Rab38* on a FHH.BN‐*Rab38* congenic line recapitulated the albuminuria phenotype (Rangel‐Filho et al., 2013). Additionally, knockdown of *Rab38* mRNA *in vitro* decreased endocytosis of colloidal gold‐coupled albumin, indicating *Rab38* modulates proteinuria by altering tubular re‐uptake but not glomerular permeability (Rangel‐Filho et al., 2013). These results from GWASs and mechanistic studies using animal models confirmed a causal role of the *Rab38* gene variants in the development of albuminuria.

Recently, Qiu et al. created an expression quantitative trait loci (eQTL) atlas for the glomerular and tubular compartments in the human kidney (Qiu et al., 2018). Integrating GWAS with eQTL, they identified the Disabled homolog 2 (*DAB2*) as a novel causal gene for CKD pathophysiology. Mutations in *DAB2* have been reported associated with CKD in several GWASs (Nair & Kretzler, 2019). Results of single‐cell RNA‐Seq of mouse kidney demonstrated that DAB2 expression is restricted to the proximal tubules and macrophages. Functional experiments confirmed that higher DAB2 levels in the kidney contributed to renal fibrosis in CKD by affecting TGFβ‐induced profibrotic gene expression.

## 8 FINAL REMARKS

Recent studies have provided a remarkable insight into new gene variants and pathways contributing to the susceptibility of hypertension‐induced ESRD. Results from these studies suggest that precision medicine should be used with hypertensive patients with different genetic backgrounds to identify the best target for blood pressure control and the choice of antihypertensive drugs. Unsolved questions that remain in this field ought to focus on how to integrate the newer genetic information with clinical practice. With breakthroughs in the future, genes contributing to renal disease have the potential to be exploited by both drug and genetic therapies to prevent and possibly even reverse hypertension‐induced kidney disease.

## CONFLICTS OF INTEREST

The authors have declared that no conflicts of interest.

## AUTHORS’ CONTRIBUTIONS

C. Z., R. J. R., and F. F. conceived the ideas in the review paper. C. Z. and F. F. participated in writing the manuscript. X. F., H. Z. W. G, and H. J. H. edited the paper. All the authors have read and approved the manuscript.
